# Pre-Clinical Evaluation of Biological Bone Substitute Materials for Application in Highly Loaded Skeletal Sites

**DOI:** 10.3390/biom10060883

**Published:** 2020-06-09

**Authors:** Sónia de Lacerda Schickert, Jeroen J.J.P. van den Beucken, Sander C.G. Leeuwenburgh, John A. Jansen

**Affiliations:** Department of Dentistry—Regenerative Biomaterials, Radboud Institute for Molecular Life Sciences, Radboud University Medical Center, Philips van Leydenlaan 25, 6525EX Nijmegen, The Netherlands; Sonialschickert@gmail.com (S.d.L.S.); Jeroen.vandenBeucken@radboudumc.nl (J.J.J.P.v.d.B.); Sander.Leeuwenburgh@radboudumc.nl (S.C.G.L.)

**Keywords:** biological bone substitute materials, highly loaded skeletal sites, animal models, biomechanical evaluation

## Abstract

The development of bone substitute materials (BSMs) intended for load-bearing bone defects is highly complicated, as biological and mechanical requirements are often contradictory. In recent years, biological BSMs have been developed which allow for a more efficient integration of the material with the surrounding osseous environment and, hence, a higher mechanical stability of the treated defect. However, while these materials are promising, they are still far from ideal. Consequently, extensive preclinical experimentation is still required. The current review provides a comprehensive overview of biomechanical considerations relevant for the design of biological BSMs. Further, the preclinical evaluation of biological BSMs intended for application in highly loaded skeletal sites is discussed. The selected animal models and implantation site should mimic the pathophysiology and biomechanical loading patterns of human bone as closely as possible. In general, sheep are among the most frequently selected animal models for the evaluation of biomaterials intended for highly loaded skeletal sites. Regarding the anatomical sites, segmental bone defects created in the limbs and spinal column are suggested as the most suitable. Furthermore, the outcome measurements used to assess biological BSMs for regeneration of defects in heavily loaded bone should be relevant and straightforward. The quantitative evaluation of bone defect healing through ex vivo biomechanical tests is a valuable addition to conventional in vivo tests, as it determines the functional efficacy of BSM-induced bone healing. Finally, we conclude that further standardization of preclinical studies is essential for reliable evaluation of biological BSMs in highly loaded skeletal sites.

## 1. Introduction

Despite the remarkable capacity of bone tissue to regenerate itself after damage, critically sized, load-bearing bone defects will not heal spontaneously without surgical intervention [1,2]. The appropriate strategy to treat such bone defects remains a clinical challenge and creates an enormous societal and economic impact [3]. Still, the use of autologous bone grafts is considered the gold standard to support the healing of large bone defects. Autografts are histocompatible, non-immunogenic and have the desired osteogenic, osteoconductive and osteoinductive characteristics [4]. However, autografts are not unlimitedly available, and their harvest often causes donor site morbidity [4,5]. In this context, the development of bone substitute materials (BSM) with a biological performance comparable to native bone and the capacity to withstand substantial mechanical loading in situ is required [6].

Among the necessary experimental routes followed for BSM development, their evaluation in a living organism (i.e., animal model) constitutes an essential requirement to establish the preclinical safety and efficacy before human clinical trials can be considered [7]. In vivo animal models allow the assessment of BSMs as a function of different loading conditions, implantation periods, tissue qualities (e.g., healthy vs. osteopenic bone) and age [8]. The preclinical translation of BSM for application in highly loaded skeletal sites has gained considerable interest during the past decades, as mimicking human non-union or delayed bone healing conditions in animal models is extremely complex. For example, the necessary type and quantity of cells, growth factors, and mechanical support required to achieve vascularization and to stimulate bone formation in highly loaded bone remain to be determined [9]. Moreover, loading patterns vary largely among different animal species and similarity to human conditions with different implantation sites is very difficult to obtain. Consequently, investigating the influence of each of these factors relies on using animal models that resemble the complex interrelations of bone healing under load-bearing conditions as closely as possible.

The current review aims to analyze the load-bearing capacity of bone tissue and to provide a comprehensive overview of the relevant biological and mechanical considerations for the design of BSMs. Moreover, the relevant aspects of preclinical animal models for the quantitative evaluation of biological BSMs intended for application in highly loaded skeletal sites are reviewed.

## 2. The Adaptive Load-Bearing Capacity of Bone

Bone is composed of nano-sized hydroxyapatite crystals, which are embedded within supercoiled assemblies of collagen type I chains. From this nanoscopic level up to the macroscopic bone structure, various levels of highly organized structural hierarchy are discerned. While the basic building blocks of bone themselves are relatively weak, their hierarchical structural organization and intimate interactions lead to remarkable mechanical properties [10,11]. Ultimately, this hierarchical organization culminates in two different types of macroscopic bone structure: (i) cortical bone, which is denser, stiffer, stronger and tougher; and (ii) cancellous bone, a more porous and mechanically ductile structure. As both bone types have different physiological functions, their mechanical properties also vary significantly (Table 1). Moreover, these mechanical properties are constantly adapting to mechanical loading. Therefore, the reference values observed in Table 1 may change according to the age of the individual human being, anatomical location and size of each individual bone, as well as the bone mineral density and the direction of the trabeculae [12].

The load-bearing capacity of the skeleton results from an adaptive functional relationship between the load a bone should sustain and its architecture. Therefore, specific features that determine the functionality of the bone itself (i.e., girth, cortical thickness, cross-sectional geometry, curvature and the number, orientation, thickness and connectivity of bone trabeculae) only develop and persist in response to continued loading [19,20,21,22]. Specific examples of such adaptation include the trabecular arrangement found in the femoral condyle [23] or the longitudinal orientation of trabeculae in vertebrae, which further allows for resistance against the predominant compressive loads [24]. This structural adaptation to load, often referred to as the anisotropic behavior of bone [25], is regulated by mechanotransduction processes by which mechanical energy is converted into signals that ultimately stimulate the remodeling of the bone [26,27,28,29].

**Figure 1:**
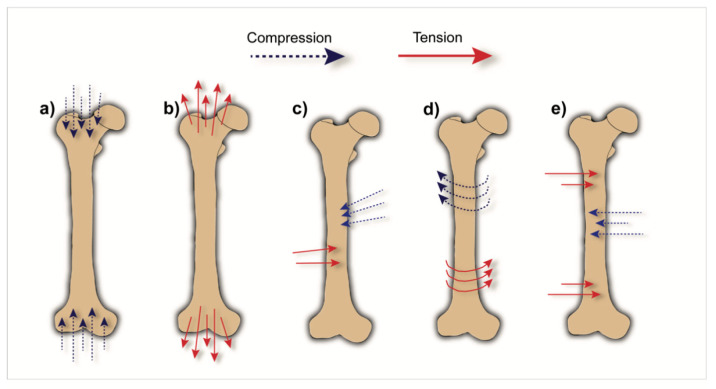
Schematic representation of common types of load acting on a bone: (**a**) compressive load; (**b**) tensile load; (**c**) shear load; (**d**) torsional load; (**e**) bending load.

Regarding the types of load that the human skeleton can experience, five different components are distinguished: (i) compression, (ii) tension, (iii) shear, (iv) torsion and (v) bending [30,31] (Figure 1). Compressive load is mainly generated by weight and gravity or by external loads applied parallel to the axis of the bone. Tensile load, on the other hand, is mainly caused by muscular activity. These loads can be applied simultaneously or sequentially. When applied simultaneously and parallel to each other, but in opposite directions, shear forces are created, e.g., within the femoral condyle, tibial plateau and pelvic region upon locomotion. Conversely, the application of loading perpendicular to the longitudinal axis of a long bone (i.e., in upper or lower limbs) results in bending stresses, in which tensile load causes convex deformation and compressive loads cause concave deformation [31]. Bending stresses occur, e.g., in the tibia, upon external rotation (i.e., the propulsive phase of walking or running), but also in the humerus due to medial-lateral movements of the arms [32].

## 3. Biomechanical Considerations for the Design of BSMs

BSMs should ideally completely fill a bone defect and provide biological stimuli and initial mechanical stability to allow for new bone formation. Subsequently, the BSM should degrade at a rate that allows for gradual load transfer from the material to the newly formed bone. At a final stage, when degradation is complete, the BSM should be fully replaced by newly formed bone of appropriate functionality [7,33,34]. However, a major challenge remains in developing and optimizing BSMs that are simultaneously load-bearing, biodegradable and osteopromotive.

The majority of BSMs that are currently applied in load-bearing skeletal regions in clinics are non-degradable. Poly(methyl methacrylate) (PMMA), for instance, is a polymer frequently used in orthopedics for procedures such as vertebral augmentation. While PMMA-based cements are innately strong and stiff to support the loads commonly experienced, e.g., in the vertebral column [35,36], they lack the capacity to degrade and remain therefore unaltered within the implantation site. In addition, the implantation of PMMA causes a redistribution of load throughout the spine, which in turn leads to loss of bone density in the vertebrae adjacent to the implantation site. In addition to non-degradability, the implantation of PMMA causes a mechanical mismatch between the implanted material and the surrounding bone (i.e., PMMA is much stronger and stiffer than native bone) that invariably leads to subsequent fractures in neighboring bone due to this stress shielding phenomenon. Consequently, long-term follow-up studies [36,37,38] confirmed that vertebrae adjacent to PMMA-filled bone fracture more frequently. Various opinions have been expressed on the importance of a mechanical (mis)match between BSMs and bone tissue. While some authors argue that the strength of the BSMs should be higher than the bone it replaces [39], clinical experience related to PMMA-based cements seems to suggest otherwise. To address this issue, the American Society of Biomechanics (ASB) has clearly highlighted the relevance of measuring in vivo loads and strains applied to bone as a basis for engineering BSMs with load-bearing capacity after implantation. Nevertheless, no consensus has yet been reached on this topic. While some authors state that BSM mechanical properties should match the mechanical properties of the defected region as much as possible [40,41,42], other studies postulate that the mechanical properties of the material should only be sufficient to allow for its handling during surgery without causing collapse and dislocation during normal activities [43].

Structurally, the optimal balance between biology and mechanics in BSMs is very hard to achieve. The presence of pores in BSMs, as well as their size, is essential to promote the ingrowth of native bone [34]. However, introducing an interconnected structure of pores within a BSM severely compromises its mechanical performance. For example, the literature demonstrates that porosity levels in the range of 80–91% result in low compressive strength (1–12 MPa) and low Young’s modulus (up to 25 MPa) values [44,45,46]. When lowering the porosity level to 70%, compressive strength can be increased to values up to 80 MPa [47,48]. In general, highly porous BSMs typically demonstrate low resistance to compressive and tensile stresses as well as a low stiffness. Additionally, the size of the pores is also of importance, as it is claimed that BSMs should possess pores of at least 100 µm, as smaller pore sizes (i.e., 75–100 µm) only allow for ingrowth of unmineralized osteoid tissue or fibrous tissue [49]. Subsequent studies have shown that pore sizes larger than 300 µm display enhanced osteogenesis, since they allow for more efficient vascularization and oxygenation of newly formed bone [50,51]. Unfortunately, the strength of a BSM typically decreases with increasing pore size [52,53].

### Biological BSMs

Over the recent years, hybrid biological BSMs have become commercially available. These consist of an osteoconductive component combined with additional compounds to enhance their biological performance [34]. Biological enhancements include the addition of cells, growth factors, and/or gene therapy. The performance of a BSM is the result of concerted biological (i.e., integration, incorporation and bioresorption) and biomechanical interactions. Improvement of the biological properties of a BSM directly translates into a more efficient integration of the material with the surrounding osseous environment, and hence a higher mechanical stability of the treated bone defect. Hybrid biological BSMs can also be combined with mechanical enhancements to further improve (either chemically and/or physically) their stability when subjected to load.

The biological improvement of BSMs has been widely explored, as evidenced by the large number of reviews that have appeared in recent years [54,55,56,57,58,59,60]. The addition of cells to BSMs is generally performed by seeding the material with the patient’s own (osteogenic stem) cells (e.g., obtained via bone marrow aspiration) [61,62,63,64,65,66] prior to or during surgical bone defect repair [67]. In addition to cell-based bone regeneration strategies, growth factors such as bone morphogenic proteins (e.g., BMP-2 and BMP-7) [63,68,69,70,71], transforming growth factor beta (TGF-β) [72], insulin-like growth factor (IGF-1), platelet-derived growth factor (PDGF) [73], or vascular endothelial growth factor (VEGF), have also been proposed. Some growth factors provide osteoinductivity, which enhances bone regeneration [56]. However, recombinant DNA technology to produce growth factors is expensive, cumbersome, and should be used with caution as the resulting growth factors are not fully similar to native equivalents, which may have a significant prospect of abnormal bone growth and even tumor formation [60,74]. For example, an FDA (Food and Drug Administration)-approved hybrid BSM consisting of a collagen sponge loaded with BMP-2 (INFUSE Bone Graft, Medtronic, Minneapolis, MN, USA) was developed in the early 2000s for spinal fusion. While the supraphysiologic therapeutic dose of rhBMP-2 causes this BSM to efficiently induce rapidly growing, mechanically stable new bone in the defect area, severe complications were reported, including uncontrolled bone formation, BMP antibody formation, bone resorption, urethra-genital complications and malignancies [75,76]. Alternatives to INFUSE Bone Graft have also been extensively researched [77]; however, the previously mentioned complications have severely hampered further R&D and clinical translation of novel carriers for BMP-2 delivery. Instead, gene therapy has been suggested to deliver genetic DNA fragments encoding growth factors by viral or non-viral methods from BSMs [58,78,79]. While the principle of using gene therapy via BSMs is similar to that of using growth factors, the main advantage of the former technique is that local cells are stimulated to produce the protein of interest in the native configuration, including its physiological glycosylation and in appropriate quantities [80].

Mechanical reinforcement of biological BSMs can be achieved by either fine-tuning physical characteristics, such as improving the morphology and/or design of the BSM (i.e., the external and/or internal architecture) [81], or by adding a reinforcing agent. This latter approach generally uses polymeric fibers added to a ceramic matrix in order to improve its flexural strength and toughness [82,83,84]. Alternatively, nano-ceramic components can be added to a polymeric matrix in order to improve its resistance to compressive loads [85,86]. Chemical alterations, such as crosslinking of polymeric structures, have also proven to successfully improve the mechanical properties of these BSMs [87,88].

## 4. Animal Models for Critical Load-Bearing Bone Defects

**Figure 2:**
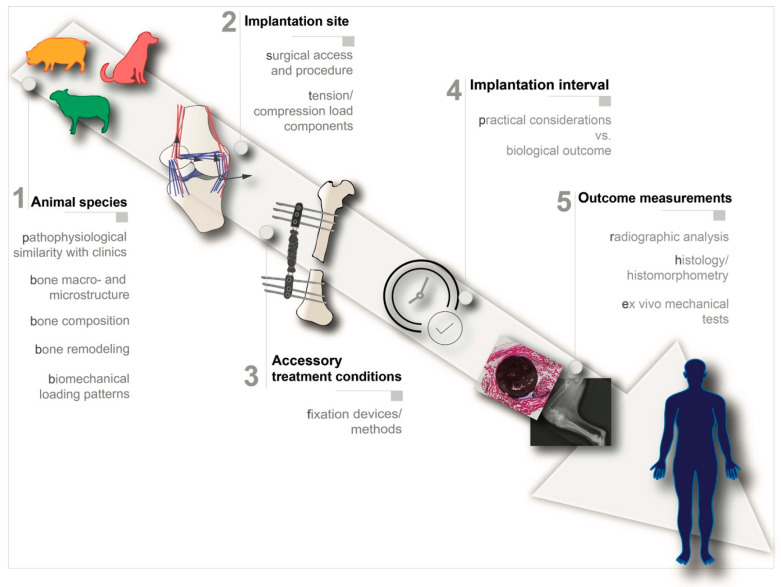
Relevant design criteria for preclinical evaluation of bone substitute materials (BSMs) intended for healing critical load-bearing defects.

A wide variety of animals has been used during the past decades for the preclinical evaluation of highly loaded bone defects, ranging from small animals such as mice, rats and rabbits, to large animals, namely sheep, dogs, pigs and cows [89,90,91]. In general, we argue that the selection of an animal model for preclinically evaluating BSMs should follow the key principles explained in the sections below [7,92,93] (Figure 2):

**1.**  **Selection of the animal species:** the selected animal species should resemble the human physiological and pathophysiological response as closely as possible;
**2.**  **Selection of implantation site:** the selected implantation site should match the clinical setting both anatomically, biomechanically and surgically;
**3.**  **Accessory treatment conditions:** the need for additional treatment conditions such as fixation devices should be carefully analyzed and mimic the real clinical intervention as much as possible;
**4.**  **Implantation period:** the implantation period should be clinically relevant;
**5.**  **Outcome measurements:** the experimental design should include concrete outcome measurement evaluations.

### 4.1. Selection of the Animal Species

Animal species are selected based on both general factors as well as more specific aspects. General factors include the cost of the purchase and maintenance of animals (i.e., housing, food and bedding), animal tolerance to captivity and ease of handling, and finally the social/ethical acceptance of using the animal experimentally [91,94]. In general, large animals are associated with more challenges regarding these general aspects than small animals. Pigs, for example, are difficult to handle and house, as they show aggressive behavior as a natural response to stress, and have, due to their size, very specific housing needs [95]. Dogs, on the other hand, are relatively easy to maintain and house, which has, in fact, led to their frequent use for musculoskeletal research until the 2000s. However, the use of dogs has markedly decreased, mainly due to ethical, emotional and legal reasons [96,97].

Specific aspects related to the selection of animal species, on the other hand, include anatomical and/or skeletal similarity, osseous macro- and microstructure, bone turnover and weight/loading patterns. In this sense, skeletally mature animals of large size and weight (e.g., pigs, dogs, sheep or goats) generally mimic the human physiological condition better than small animals [90,92,98]. Nonetheless, the use of small animals (i.e., rats or rabbits) is acceptable for the preliminary biomechanical assessment and initial validation of load-bearing models but should be followed by more clinically relevant animal models [92,99]. Detailed comparisons between pig, dog, sheep and goat models have been extensively described in the literature [92,94,98,100].

#### 4.1.1. Anatomic Analogy and Bone Macro- and Microstructure

Pigs are often regarded as the most suitable animal model for biological evaluation of bone healing and bone remodeling, as their remodeling capacity and bone turnover is nearly identical to humans [101]. In addition, their bone morphology, anatomy and structure resemble human bone closely, even though the trabecular network is slightly denser [102]. Special care should be taken when selecting the breed of pigs, as the vast majority of pig breeds often have excessive body weight and tend to have very high levels of bone density, and hence decreased structural similarity to human bone. Consequently, load-bearing BSM research has also been conducted in mini- and micro-pigs [103,104], as these animals are still very similar to the human condition but weigh less than common pigs [95].

Dogs and humans are qualitatively similar in terms of composition and bone mechanical properties [89,105]. Nevertheless, dog and human bones are slightly different regarding bone microstructure and remodeling, as dogs possess a combination of secondary bone structure with plexiform bone. This type of bone is commonly found in fast-growing animals and is characterized by a special bone architecture that provides bone stiffness and strength within a relatively short period of time, while allowing for slow deposition of mature bone.

Sheep and goats in their mature state possess a body morphology (i.e., weight and size) and the specific dimensions of long bones very similar to adult humans. Microstructurally, both sheep and goats possess plexiform bone combined with primary bone. At an early stage of development, bone density in sheep is generally highly similar to humans [106]. As these animals age, remodeled secondary osteonal bone becomes more prevalent and the density increases to levels higher than in humans. Subsequently, skeletally mature sheep possess a higher bone strength than human bones [106]. Generally, bone mineral composition [107], as well as the bone turnover and remodeling capacity [96] of both sheep and goats are comparable to humans.

#### 4.1.2. Weight/Loading Patterns

Load distribution patterns should be considered in the process of animal selection, as animals experiencing similar mechanical stresses in homologous anatomical locations to humans are more likely to resemble the loading conditions experienced in humans. The distribution of loads is mainly influenced by the body weight in combination with the type of gait. Body weight influences the extent of biomechanical loading [108], and consequently goats or sheep, which have body weights comparable to humans, are often selected in studies testing the biomechanical performance of bone substitutes. Regarding the type of gait, dogs, goats, sheep or pigs exhibit a quadruped gait, i.e., use four limbs for locomotion, in contrast with humans that have a bipedal gait and use two limbs. Consequently, the load in a quadruped is distributed in a different way than in humans. Taylor et al. [109] compared the quadruped nature of sheep with the bipedal gait of humans and concluded that the loading of the hind limb bones of sheep is roughly half of the load observed for humans upon walking.

**Figure 3:**
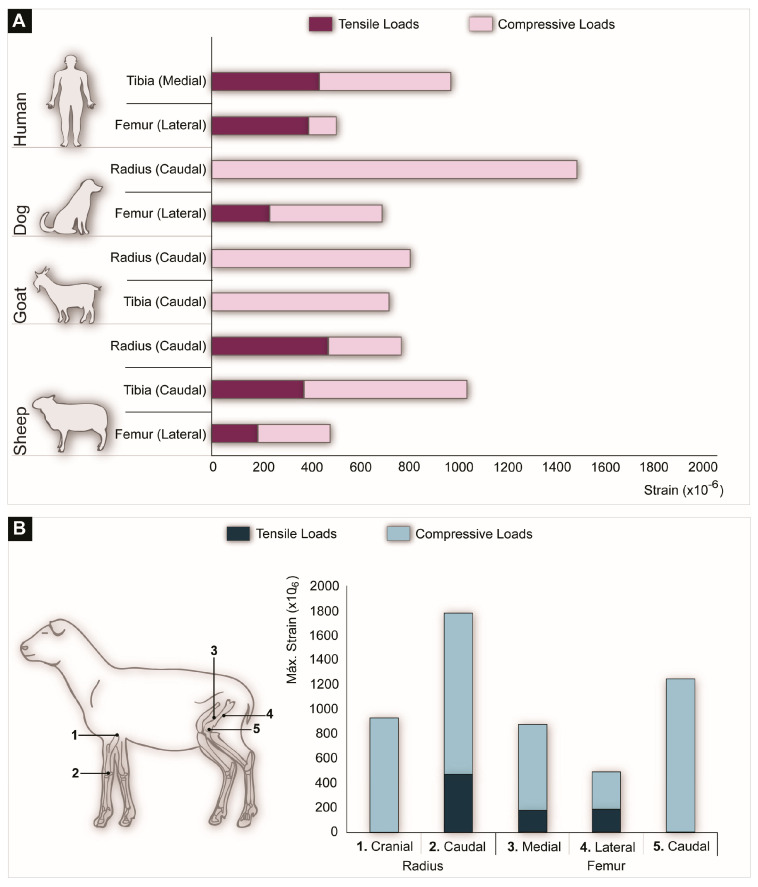
(**A**) Strain values obtained by implanting a strain gauge at different anatomical locations in different animals as compared to humans during walking. (**B**) In vivo strain measurements obtained by strain gauges at different locations on the radius and the femur of a sheep walking at a speed of 1 m/s. The strain gauge detected compression stresses dominating in the cranial aspect of the radius, as well as the caudal aspect of the femur. In contrast, the caudal aspect of the radius and the medial and lateral aspects of the femur were subjected to both compressive and tensile stress (the data used for this figure were compiled from [19,21,110,111,112,113,114,115,116]).

Through the implantation of in vivo strain gauge devices, mechanical straining can be assessed locally. Since strain gauges measure bone deformation, these devices allow for the detection of compressive and tensile stresses. In vivo strain measurements performed at different anatomical locations of humans and animals during walking are presented in Figure 3. Figure 3A shows that at homologous anatomical locations, such as the femur or the tibia, compressive and tensile strains vary to a large extent, both between humans and animals and within different animal species. In addition, Figure 3B demonstrates the load variation between the front limbs and the back limbs of a sheep.

### 4.2. Selection of Implantation Site

**Figure 4:**
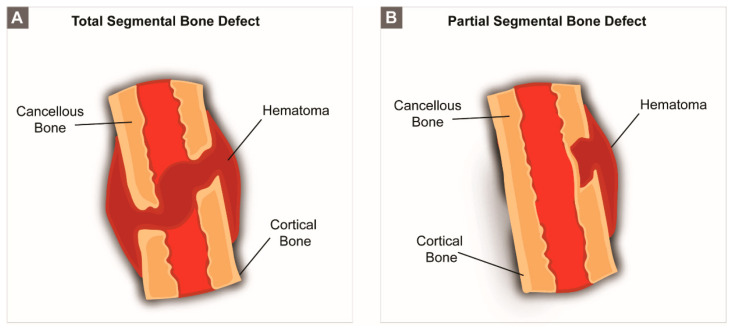
Schematic representation of (**A**) a full segmental bone defect and (**B**) a partial segmental bone defect.

**Limb bones**. *Segmental bone defects*. Clinically, segmental bone defects have a non-union rate as high as 21% [117] and originate from trauma or resection of necrotic and/or infected bone. In humans, the most commonly used anatomic region affected by segmental bone defects is the tibial shaft [98]. Critically sized segmental defects may include an entire segment of bone (Figure 4A) or just the cortical component (Figure 4B). Limb bones are highly loaded, not only due to weight support (i.e., in the case of lower limbs), but also because these structures are constantly subjected to tensile stresses that originate from the action of muscles, tendons or ligaments.

Preclinical animal model studies focusing on the assessment of biological BSMs in critical-sized segmental bone defects have been performed by means of entire or partial segmental bone defect models, using the ulna, tibia, radius and femur of mini-pigs, dogs, goat and sheep (Table 2). Ovine models are among the most often selected animals. To this end, the tibia is the major weight-bearing bone of lower leg [98] and, therefore, the most commonly used anatomical site [100].

Critical-sized segmental defects are generally defined by considering the diameter of the shaft multiplied by factor 2.0–2.5 [120,121], although some studies report the use of defects three times larger than the diameter of the bone [120]. The creation of such defects is usually performed by an oscillating saw, although the use of Gigli wire saws [122], motorized dental drills [123] and CO_2_ lasers [124] has also been reported. While creating the defect, special care should be taken in removing the periosteum, as its presence can potentially affect the critical nature of the defect and facilitate spontaneous healing [98,120]. Finally, the creation of a segmental bone defect in a limb bone weakens the entire structure severely and the placement of a biological BSM alone might not be sufficient to ensure mechanical stability for bone regeneration. Like the clinical situation, segmental bone defects often require the use of fixation devices. For segmental defects that include a full segment of bone, these devices will reduce movement between the bone extremities. In the case of defects that only include the cortical bone, fixation devices will act as a cantilever by counteracting bending stresses. The types and applications of specific fixation devices will be further discussed in Section 4.3.

**Figure 5:**
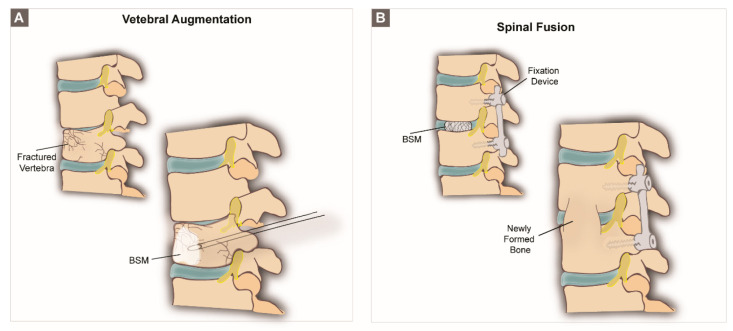
Schematic representation of (**A**) a vertebral augmentation procedure and (**B**) a spinal fusion procedure.

**Spine**. *Vertebral models*. The spine of both humans and animals is a heavily loaded skeletal structure composed of a series of freely hinged vertebrae to which axial compressive load originating from weight support and gravity is counterbalanced by tensile loads originating from muscles and ligaments [125]. In clinics, two surgical procedures are often performed for bone regeneration in the spinal region: vertebral augmentation (Figure 5A) and spinal fusion (Figure 5B). Vertebral augmentation aims to treat vertebral compression fractures (VCFs) by reinforcing and stabilizing fractured vertebral bodies using PMMA-based cement through a minimally invasive percutaneous injection [126,127]. However, as the use of PMMA is associated with several drawbacks, extensive preclinical research has been performed to simulate vertebral augmentation procedures and test new, alternative vertebral cements (Table 3). Spinal fusion procedures, on the other hand, are clinically performed in the cervical, lumbar and/or thoracic spine. This technique aims to fuse two or more vertebrae together in order to treat medical conditions that arise from degenerative, traumatic and oncologic pathologies. However, pseudarthrosis or failed fusion rates are reported to be as high as 40% in primary spinal fusion surgery and up to 60% in revision cases [128], which leads to the need for the improvement of new spinal fusion materials and their evaluation through preclinical models (Table 4).

Several studies have shown striking similarities between the spine of humans and quadrupeds, not only in the osseous structural arrangement (suggesting that quadruped and biped spines experience similar loads), but also in mechanical properties of specific spinal segments [133,134]. Nonetheless, it should be mentioned that the axial compression stress experienced in quadrupeds is higher than in humans, which limits the transferability of the results of animal experiments to the human situation [125]. In addition, the shape of the vertebral bodies of virtually all domestic animals is quite different to those of humans [135].

Mini-pigs, dogs and goats have been used as preclinical models in vertebral augmentation, but the used protocols vary considerably, as well as the selected surgical approach and vertebral defect characteristics. Recent trends demonstrate that (i) sheep are the animal species of choice for preclinical vertebral augmentations, and (ii) these procedures are mainly performed through minimally invasive, standardized techniques [35,84,127]. However, in sheep, the surgical access of the vertebrae is often complicated by the large muscle mass in the lumbar area, the size of the transverse processes, the different orientation of the facet joints and mainly the slim and hour-glass shaped vertebral bodies (i.e., slightly different from humans) [135]. For this reason, preclinical vertebral augmentations should be performed under fluoroscopic guidance.

The basic requirements for preclinical studies mimicking spinal fusion procedures depend on the selection of animal species with parallel and sufficiently large vertebral endplates [136]. Canine models have been often utilized for mimicking lumbar fusion experiments [130], although some authors also considered this model for cervical and thoracic fusions [131]. Conversely, goat models are only used to study cervical fusions [132]. Finally, in sheep, the lumbar spine is frequently studied [78], whereas the thoracic spine and cervical spine are less often studied. Pigs are more often selected to study spinal fusion techniques (i.e., operative procedure, fixation placement, etc.) rather than preclinical experimentation of new biological BSMs for spinal fusion applications.

### 4.3. Acessory Treatment Conditions

**Fixation devices.** Due to the heavily loaded nature of many critically sized skeletal defects, fixations devices are often required in experimental animal studies. Fixation techniques are generally performed externally or internally and have been extensively reviewed in the literature [98,137,138].

External fixation is a versatile method, often reported for segmental bone defects, in which pins are placed widely separated within bone fragments. These pins are screwed into the bone and subsequently connected by a rod outside the body. Since the stabilizing devices are located outside the body, this technique provides an open space for the implantation of the selected biological BSM. On the other hand, due to the creation of a percutaneous exit-site, infections of the pin track can occur. In addition, the pins can loosen during the healing process, resulting in an unstable defect site [139]. Also, internal fixation devices, such as intramedullary nails and plate fixators, have been used to stabilize and join segmental bone defects. Intramedullary nails consist of a metal rod inserted into the medullar cavity of the bone. Since the intramedullary nail is placed in the center of the bone, it is able to tolerate the applied stresses due to weight in a parallel direction, thereby ensuring mechanical stabilization and avoiding axial deviation of the components of the fixation device. Intramedullary nails should nonetheless be used with caution, as they have been associated with the impairment of blood circulation and thermal necrosis [140]. In addition, the size of the nail might severely limit the space available to apply the biological BSM.

Plate fixators are extensively utilized, both for segmental bone defects and spinal procedures. In this technique, the selected plates, which might be metallic or polymeric in nature, are tightly screwed to the edges of the defect and remain attached to the bone surface for a determined period of time. In segmental bone defects, plate fixation has a minimal influence on the defect and provides space for the implantation of biological BSMs. However, since the plates carry the load in an eccentric manner, this fixation method is prone to axial deviations, causing either failure of the BSM or healing in an erroneous direction [141,142]. In the spine, these fixation devices are only utilized in the case of pronounced vertebrae mobility to ensure an accurate fusion between vertebrae.

### 4.4. Implantation Period

To determine if a biological BSM succeeds to regenerate bone under the influence of physiological loading, the implantation period should be sufficiently long to allow for both bone formation and remodeling. Since each animal species requires a different period of time for bone regeneration, the implantation period depends on the selected animal. Another important aspect is the age of the animal and the presence/absence of an artificially induced osseous pathological condition (i.e., osteoporosis), as both these factors influence bone healing. ASTM standards [121], for example, recommend implantation intervals longer than 12 weeks for skeletally mature (i.e., >15 months old), healthy ovine models. Dogs display faster bone remodeling rates than sheep and require therefore shorter implantation periods. Pigs, which have a bone formation process comparable to humans, require 6 months to 1 year to achieve complete healing of a critically sized defect.

In general, however, the majority of the available studies rely on follow-up periods that do not exceed six months, which has previously been considered too short to evaluate long-term effects of BSMs on bone regeneration and remodeling as well as BSM degradation [98]. Nevertheless, it should be emphasized that the selected implantation period is often compromised by practical aspects regarding costs and ethical concerns. Consequently, researchers tend to select the shortest implantation period as possible to answer the specific research question under investigation.

### 4.5. Outcome Measurements

In general, the repair of large, load-bearing bone defects is analyzed in vivo by various outcome measurements, all of which focus on the characterization of the former defected region in terms of bone formation and functionality relative to the pre-defect condition. To this end, several techniques have been employed, both during and after animal experiments to allow for the monitoring of bone healing and regeneration [143,144,145]. Monitoring healing progression in vivo can be performed using fracture stiffness measurements via, e.g., goniometers or transducer fixators [143]. Further, radiographic and computed tomography (CT) or cone beam CT (CBCT) have been applied for measuring bone volume and mineralized tissue formation and density [142,145]. In addition, histological techniques are commonly employed to evaluate: (a) mineralized tissue formation, (b) integration with the host, (c) cellular components such as marrow and vasculature, and (d) the host inflammatory response to the BSM. Together, these techniques can provide a comprehensive assessment of the regenerated bone and its effectiveness in mitigating bone loss [100]. Nonetheless, the functional capability and structural integrity of both the newly formed and surrounding bone cannot be assessed solely through the previously mentioned techniques [144], for which ex-vivo mechanical testing of the freshly explanted bone samples is essential and arguably the most important outcome measurement [92,98].

Ex-vivo mechanical tests rely on the comparison between the mechanical properties of an intact bone structure (i.e., control) vs. an explanted experimental bone structure. Four classical biomechanical tests are commonly used for the assessment of these biomechanical properties, i.e., tension, compression, bending and torsion tests. Bending and torsion combine both compression and tension and allow for a more complete assessment of the mechanical properties of the evaluated bone [52]. Table 5 lists the biomechanical tests that are reported in literature as well as their respective advantages and disadvantages.

Ex-vivo mechanical tests may be performed on either entire bones or partial bone segments containing the implanted BSM. Entire bones are usually tested without further processing, whereas partial bone segments are machined into a specific shape to comply with the requirements of specific type of mechanical test. This methodology results in specimens with identical dimensions, which decreases the possible experimental errors that may arise from shape discrepancy between samples. However, it should be recognized that machining a specimen harvested from an animal might alter the properties of the bone, Therefore, when machining these specimens, special care should be taken to minimize damage to the specimen by, e.g., constant cooling during cutting and/or machining in s frozen state. Additionally, ex-vivo mechanical tests should be performed as soon as possible after explantation to avoid excessive drying of the specimens. Immediately after harvesting, bone specimens should be stored in ice. It has been demonstrated that bone specimens kept at room temperature for 24 h lost 3% of their Young’s modulus [146]. If long-term storage cannot be avoided, bone tissue should be frozen and kept hydrated (to avoid freeze drying), either by maintaining the surrounding musculature or by wrapping the structure in gauzes soaked in saline solution. Submersing the bone specimen in embalming solutions such as formalin or alcohol as a preservation method should be avoided, as these solutions might change the mechanical properties of bone [147].

## 5. Progress in the BSM Field—Clinical Translatability

In recent years, the bone substitute field has evolved from the traditional one-way “bench-to-bedside” into an interactive “bench-to-bedside-and-back-again” approach [148]. The driving forces behind this interactive back-and-forth approach are currently unmet clinical needs. A precise understanding of current clinical practice is therefore required in order to identify specific clinical needs that are yet to be addressed and ultimately lead to an adequate balance between technology-push (i.e., the development and fine tuning of a product) and market-pull (i.e., the need/requirement for a new product/treatment). Equally important to the clinical need are additional practical factors, such as ethical and regulatory issues (both on institutional and governmental levels), funding issues for product development, product upscaling and production, and aspects related to physician acceptance of a new treatment method. Failing to consider all these aspects will cause BSMs to fail to reach the clinical setting despite outstanding performances at the preclinical level. A good example of such failure are scaffold-based bone substitute therapies, from which the translation to clinical has still not been achieved despite 25 years of research, research funding totaling hundreds of millions of dollars, and over 12,000 research papers published in the past 10 years alone [149,150]. Ceramic scaffolds, for example, have demonstrated excellent preclinical results over the years [151,152]. Nonetheless, these materials are expensive and cumbersome to produce and upscale, difficult to use in clinics, and subject to multiple regulatory conditions (especially when containing hybrid matrixes or living cells). Therefore, the ability to bring clinical requirements associated with the practical aspects of its clinical application earlier into the development process of a BSM is a critical requirement for investigators, study sections and funding agencies to efficiently ease the path to clinics.

## 6. Closing Remarks

Nowadays, newly developed biological BSMs reflect the concept that biological and mechanical properties can work synergistically and ultimately allow for a degradable, mechanically stable and osteocompatible BSM to successfully regenerate highly loaded bone defects. Preclinical surrogate models play a vital role in the continuous optimization of bone regenerative technologies. Nonetheless, mimicking human load-bearing bone defect conditions is extremely complex and requires a detailed planning of the selected preclinical models. In fact, the translational value of a preclinical animal model is strictly dependent on the design of the selected model and its influence in the obtained outcome. For this reason, although challenging, animal models should resemble the clinical course of the human indication for a BSM as closely as possible. Further, preclinical studies should be systematically standardized into predefined protocols, facilitating direct comparisons between the outcome of different BSMs evaluated under the same preclinical experimental conditions. Finally, it is also critical that the access to large animal models is made available for the entire scientific community, as well as the expertise necessary to perform such animal studies successfully. Unfortunately, while the number of bioengineers, biomaterial scientists, molecular and cellular biologists working in the BSM field continues to increase, the number of research groups which have the expertise, infrastructure and track record of well characterized and validated large preclinical animal models continues to decrease inexorably.

## Figures and Tables

**Table 1:** Mechanical properties of human bone.

Parameter	Mechanical Characteristics ^1^	
	Cortical Bone	Cancellous Bone
Compressive strength (MPa)	70.0–200.0	0.1–30.0
Tensile strength (MPa)	90.0–170.0	10.0–20.0
Flexural strength (MPa)	135.0–193.0	10.0–20.0
Ultimate strain at fracture (%)	1.0–3.0	5.0–7.0
Elastic modulus (GPa)	3.0–30.0	0.1–5.0
Porosity (%)	5.0–30.0	50.0–95.0

^1^ Values compiled from [11,13,14,15,16,17,18].

**Table 2:** Selection of preclinical segmental bone defect studies for development of biological BSMs.

Animal			Bone	Segmental Bone Defect			BSM	Fixation Method	Time-Points (Weeks)	Outcome Measurements	Ref.
Type	Species	Weight (Kgs)		Type	Dimensions (cm)	Method of Production					
Pig	Yucatán mini-pigs (Sus scrofa)	37.0 ± 3.6	Tibia	Partial segmental defect	1	Oscillating bone saw	Collagen scaffold/microbubble-enhanced BMP6 plasmid	Internal fixation (custom-made six-hole LC-DCP plates)	1, 2 and 3	Protein expression analysis, µCT scan, histology and histomorphometry and ex-vivo mechanical test (i.e., torsional)	[79]
	Mini-pigs (Sus scrofa domesticus)	N.I.	Femur	Total osseous mid-diaphyseal defect	1.5	Oscillating bone saw	Nanocomposite scaffold HaP/collagen/BMSCs	Internal fixation (LC-DCP plates (4.5 mm-thick) fixed with four cortical titanium locking screws (diameter: 4.5 mm)	16	Plain X-ray, µCT scan, histology and histomorphometry	[64]
Dog	Mongrel dogs (Canis lupus familiaris)	30.3 ± 8.6	Radius	Total osteoperiosteal middiaphyseal defect	2.5	Oscillating bone saw	rhBMP2/collagen sponge carrier	External fixation	24	Plain X-ray, histology and histomorphometry and ex-vivo mechanical test (i.e., torsional)	[68]
	Mongrel dogs (Canis lupus familiaris)	4.5 ± 0.5	Femur	Total osteoperiosteal middiaphyseal defect	1.1	Oscillating bone saw	PCL bread scaffolds, PCL bead scaffold/BMP2	2.0 mm Intramedullary pin and 2.7 mm universal locking plate	4, 8 and 24	Plain X-ray, serum chemistry, histology and histomorphometry and RT-qPCR	[69]
Goat	N.I.	19.6 ± 3.4	Femur	Total osteoperiosteal mid-diaphyseal cortical defect	2.5	Oscillating bone saw	Coral cylinder/BMSCs	Internal fixation rod and interlocking nails	16 and 32	Plain X-ray, histology and histomorphometry and ex-vivo mechanical test (i.e., three-point bending)	[66]
Sheep	North-Holland and black-faced sheep (Ovis aries)	54.2 ± 7.6	Tibia	Total osseous total mid-diaphyseal defect	3	Oscillating bone saw	Granular porous HaP/rhOP-1, Granular porous HaP/autologous bone marrow aspirate	Intramedullary nail	12	Plain X-ray, histology and histomorphometry and ex-vivo mechanical test (i.e., torsional)	[118]
	German blackheaded mutton sheep (Ovis aries)	68.1 ± 8.4	Metatarsus	Total osseous mid-diaphyseal defect	2	Oscillating bone saw	Titanium (Ti6Al4V) implants/collagen/β-TCP	Internal fixation (LCP 3.5 mm-thick, stainless steel, 8-holes)	12 and 24	Plain X-ray, µCT scan, BMD and ex-vivo mechanical test (i.e., torsional)	[119]

N.I.: Not Indicated; LC-DCP: Dynamic compression plates with limited bone contact; µCT: Micro-computed tomography; DEX: Dual-energy X-ray absorptiometry; HaP: Hydroyiapatite; BMSCs: Bone marrow stromal cells; BMP: Bone Morphogenic Protein; TCP: Tricalcium phosphate; RT-qPCR: Quantitative reverse transcription polymerase chain reaction; rhOP-1: Recombinant human osteogenic protein-1; LCP: Locking compression plate.

**Table 3:** Selection of preclinical vertebral augmentation studies for biological BSM development.

Animal				Vertebral Defect			BSM	Time-Points (Weeks)	Outcome Measurements	Ref.
Type	Species	Osteoporotic/Osteopenic Condition	Average Weight (kg)	Selected Vertebral Segments	Defect Size (Diameter × Depth)	Surgical Technique				
Pig	Piétrain (Sus scrofa domesticus)	No	N.I.	L3	10 × N.I. mm	N.I.	TCP, TCP/rhBMP7, TCP/autologous bone marrow aspirate	4	Plain X-ray, ex-vivo mechanical test (i.e., compression)	[70]
Goat	Domestic goat (Capra aegagrus hircus)	Yes	17.0 ± 1.5	L2 and L4	5 × 10 mm	Lateral retro-peritoneal exposure of spine	rhBMP2/GM/CPC, rhBMP2/CPC	6 and 16	µCT scan, DEX, histology and histomorphometry, ex-vivo mechanical test (i.e., push-out/compression)	[71]
Sheep	Merino sheep (Ovis aries)	Yes	90.9 ± 10.7	L1, L4, L5	5.0 × 14.0 mm	Fluoroscopy-guided minimally invasive ventrolateral approach	CPC/PLGA fibers, CPC/PLGA fibers/BMP2	12 and 36	Plain X-ray, µCT scan, DXA, histology and histomorphometry, mechanical testing (i.e., compression)	[83,84]
	Swiss alpine sheep (Ovis aries)	No	72.6 ± 16.4	C3–C5	2.8 × N.I. mm	Fluoroscopy-guided minimally invasive ventral approach	Fs/SrCo3, Fs/SrCo3/PTH.	16	Plain X-ray, µCT scan, histology and histomorphometry	[129]

N.I.: Not Indicated; PPF: Poly(propylene fumarate); TCP: Tricalcium phosphate; HaP: Hydroxiapatite; TtCP: Tetracalcium phosphate; DCP: Dicalcium phosphate; µCT: Micro-computed tomography; CaP: Calcium Phosphate; GM: Gelatin microparticles; DEX: Dual-energy X-ray absorptiometry; rt-PCR: Reverse transcription polymerase chain reaction; Fs: Fibrin scaffold; SrCO3: Strontium Carbonate; PTH: Human parathyroid hormone.

**Table 4:** Selection of preclinical spinal fusion studies for biological BSM development.

Animal			Spinal Fusion			BSM	Fixation Method	Time-Points (Weeks)	Outcome Measurements	Ref.
Type	Species	Average Weight (kg)	Vertebral Segment	Method of Vertebrae Dislocation	Surgical Approach					
**Dog**	Beagle (Canis lupus familiaris)	14.5 ± 0.5	L1/L2 and L4/L5	Vertebrae were decorticated by high speed burr	Posterolateral approach	BCP, BCP/rhBMP2 and BCP/AB204	N.U.	8	Plain X-ray, µCT scan, manual palpation, histology and histomorphometry	[130]
	Beagle (Canis lupus familiaris)	10.5 ± 1.5	T9/T10	No dislocation, only curetting of the anterior longitudinal ligament and intervertebral disc	Anterolateral approach	RhBMP2/PLA-PEG	N.U.	4, 8 and 12 months	Plain X-ray, µCT scan, manual palpation, histology and histomorphometry	[131]
**Goat**	N.I.	N.I.	C3/C4	Anterior discectomy	Right anterolateral approach	Hat shaped titanium cervical intervertebral fusion cage coated with HaP, IGF-I and TGF-β1	N.U.	1, 2, 4, 8, 12	Plain X-ray, ex-vivo mechanical test (i.e., compression and bending), histology and histomorphometry	[132]
**Sheep**	Texas/Gotland breed sheep (Ovis aries)	715 ± 15.5	L2/L3 and L4/L5	Vertebrae were decorticated by high speed burr	Posterior approach	i-Factor™ Flex (ABM+P-15)	N.U.	18	µCT scan, histology and histomorphometry	[78]

BCP: Biphasic calcium phosphate; AB204: Activin A/BMP2 chimera; N.U.: Non-utilized; HaP: Hydroxiapatite; IGF-I: Insulin-like growth factor 1; TGF: Transforming growth factor; ABM+P-15: Anorganic bovine-derived hydroxyapatite matrix + synthetic 15 amino acid sequence.

**Table 5:** Types of ex-vivo mechanical tests for the evaluation of the biomechanical properties of explanted bone specimens.

Mechanical Test	Schematic Representation		Advantages	Disadvantages	Observations
Tensile test	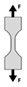	Specimen is usually a round bar with a reduced middle region and a length-to-diameter ratio of 5:1.	Allows for relatively easy assessment of the strain of bone (by using strain gauges).	1. Usually requires large specimens; 2. Some bending might be applied to the specimen, leading to measurement errors; 3. Requires the specimen to be machined; 4. Only one component of load is applied—incomplete evaluation of the mechanical properties.	1. Easier to perform for cortical bone than cancellous since cancellous bone is difficult to clamp; 2. Tensile load is calculated by dividing the applied force divided by the cross-sectional area in the midsection of the specimen.
Compression test	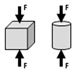	Specimen is usually a cube or cylinder having a length-to-diameter ratio of 2:1.	1. Usually requires small specimens; 2. Fabrication of the specimens is easier than for tensile tests.	1. The presence of “end effects”^1^ often leads to errors; 2. Strain is very difficult to measure; 3. Only one component of load is applied—incomplete evaluation of the mechanical properties.	Reducing the size of the specimen increases the risk of “end-effects” ^1^.
Bending test	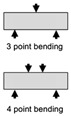	Can be performed in a 3- or 4-point bending set-up.	Both components of load are applied—tensile stresses are present on one side of the specimen and compressive stresses on the opposite side.	1. Highly influenced by the size and shape of the specimen—defects throughout the specimen may lead to non-accurate results; 2. A 3-point bending produces several transverse shear stresses in the middle of the specimen while 4-point bending model applies almost pure bending stresses.	1. Since bone is weaker in tension than compression, failure usually occurs on the tensile side of the bone; 2. Positioning of the specimen should be very precise, since each loading point has to be equal to obtain accurate results.
Torsion test	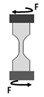	Specimen has a reduced central portion to ensure that the failure occurs in the middle part.	1. Measures the biomechanical properties of bone under shear stress; 2. When the specimen is twisted, shear stresses vary from zero at the center of the specimen to the maximum value at the surface; 3. Both compression and tension are present.	1. Requires the specimen to be machined; 2. Practical issues may occur (i.e., clamping the sample to the testing device).	Testing strongly influenced by the shape of the specimen.

^1^ “End effects” are measurement errors that originate from the damage incurred at the end surfaces of machined specimens.
